# Supplementary material for: Exosomal cargo including microRNA regulates sensory neuron to macrophage communication after nerve trauma
Source: Nat Commun. 2017 Nov 24;8:1778. doi: 10.1038/s41467-017-01841-5 (PMC5701122; doi:10.1038/s41467-017-01841-5)
Supplement: Supplementary file 1 — Supplementary Information [file 41467_2017_1841_MOESM1_ESM.pdf]

Supplementary information

Supplementary Tables

Supplementary Table 1. LNA-based miR-21-5p inhibitor and the relative scrambled control (mismatch sequence) were custom designed by Exiqon.

/5FAM/ indicates that the oligo is labeled with FAM at the 5’ end. The asterisk * indicates a phosphorothioate bond, which is an oligo backbone modification needed for *in-vivo* stability.

Oligo_name	Sequence
miR-21-5p antagomir	/5FAM/T*C*A*G*T*C*T*G*A*T*A*A*G*C*T
Scrambled oligomer	/5FAM/T*C*A*G*T*A*T*T*A*G*C*A*G*C*T

Supplementary Table 2. Sequences of miRNAs analysed in this study provided by Exiqon.

ID-miRBase Version 19.0	Accession number	Mature sequence 5’-3’
mmu-miR-21a-5p	MIMAT0000530	UACUUAUCAGACUGAUGUUGA
mmu-let-7b-5p	MIMAT0000522	UGAGGUAGUAGGUUGUGUGGUU
mmu-miR-124-3p	MIMAT0000134	UAAGGCACGCGGUGAAUGCC
mmu-miR-134-5p	MIMAT0000146	UGUGACUGGUUGACCAGAGGGG
mmu-miR-155-5p	MIMAT0000165	UUAAUGCUAAUUGUGAUAGGGGU

Supplementary Table 3. Sequences of primers for real-time PCR analysed in this study and provided by Sigma Aldrich.

Target gene	Forward primer	Reverse primer	Accession Number
Actb	5'- GGCTGTATTCCCCTCCATCG	5'- CCAGTTGGTAACAATGCCATGT	NM_007393.5
Arg1	5'-GTGAAGAACCCACGGTCTGT	5'-CTGGTTGTCAGGGGAGTGTT	NM_007482.3
Dicer1	5'- GATGCAGCCTCTAATAGAAAAG	5'- CTGTAGCTCCGGCCAACAC	NM_148948.2
Gapdh	5'-ACTCCACTCACGGCAAATTCAACGG	5'-AGGGGCGGAGATGATGACCC	NM_001289726
Mrc1	5'-CAGGTGTGGGCTCAGGTAGT	5'-TGTGGTGAGCTGAAAGGTGA	NM_008625.2
Nos2	5'-GGCAAACCCAAGGTCTACGTT	5'-CTCAAGTTCAGCTTGGT	NM_010927.4
Rela	5'-CTTGGCAACAGCACAGACC	5'-GAGAAGTCCATGTCCGCAAT	NM_009045.4
Spry2	5'-GGTTGGTGCAAAGCCGCGAT	5'-AGGGCATCTCTTGGATCCGGC	NM_011897.3

Supplementary Figures

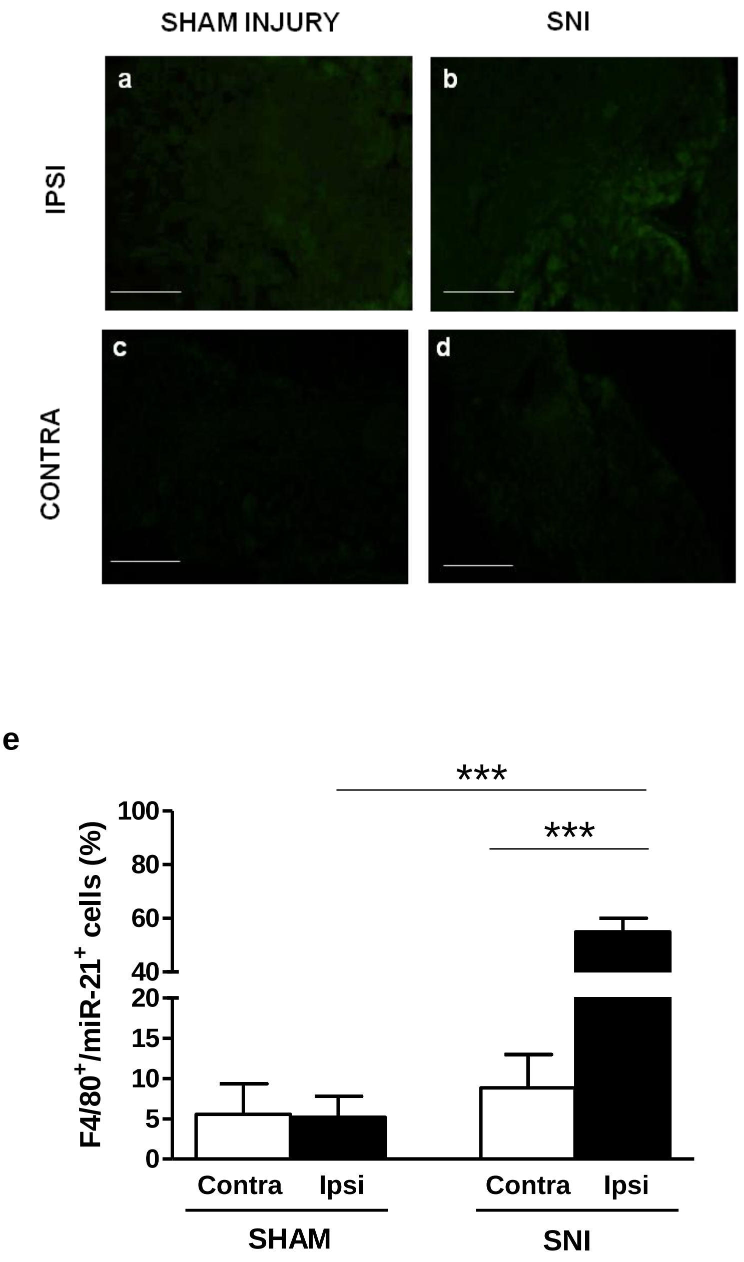

Supplementary Figure 1. No miR-21 expression is detected in DRG using scrambled probe. F4/80$^+$/ miR-21$^+$ cell number is increased in DRG neurons following SNI injury. (**a-d**) miR-21 scrambled probe expression by fluorescence *in situ* hybridisation (FISH) in ipsilateral, contralateral L5 DRG neurons 7 days after sham and spared nerve injury (SNI). Scale bar = 100 µm. (**e**) Quantification of F4/80$^+$ cells (macrophages) that also express miR-21 in L4/5 DRG. Data are means ± S.E.M., n=3 mice/ group. ****P*<0.001, one-way ANOVA, post-hoc Bonferroni.

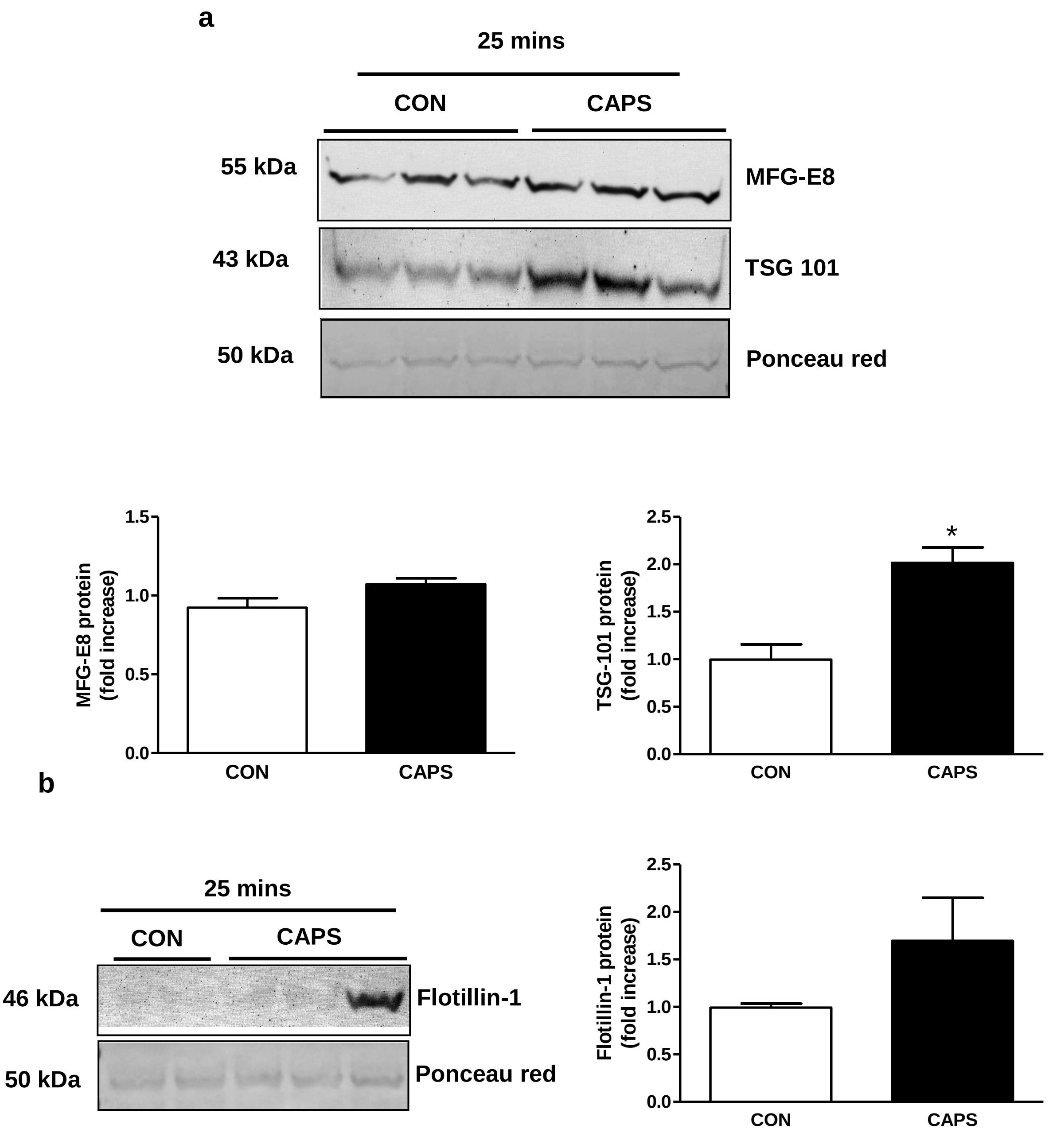

Supplementary Figure 2. Short incubation of DRG neurons with capsaicin induces a significant increase in the expression of the exosomal marker TSG101, but not MFG-E8 and Flotillin-1. Representative Western blot and quantification of protein expression for the exosomal markers MFG-E8, TSG101 (**a**) and Flotillin-1 (**b**) in the culture media of DRG neurons incubated with HEPES buffer+glucose (1mg/ml) (CON) or Capsaicin (1 µM; CAPS) for 25 minutes. Data are means ± S.E.M., n=3 cultures; *P<0.05, Student's t test.

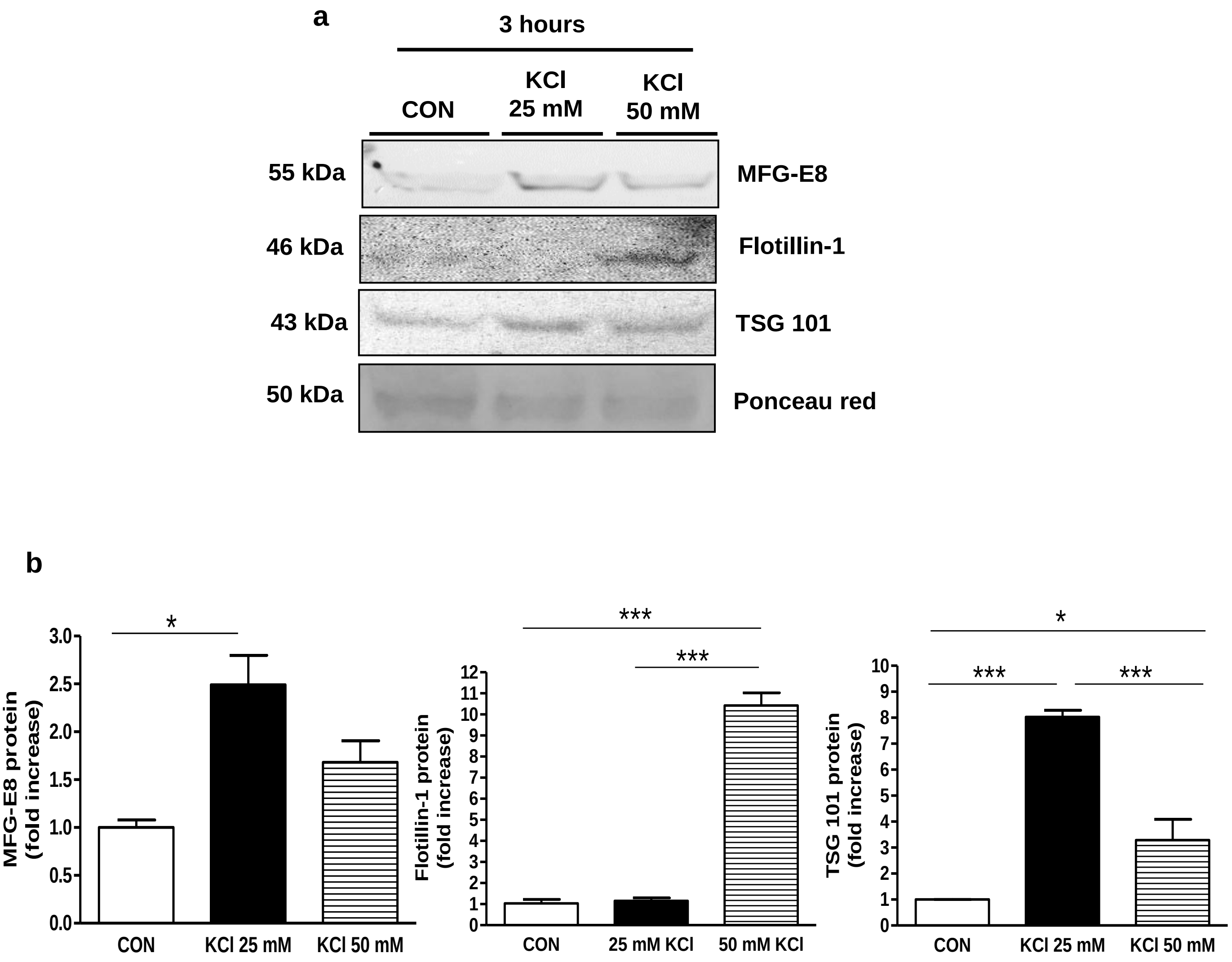

Supplementary Figure 3. DRG neurons in culture release exosomes following potassium chloride incubation. (a) Representative Western blots and **(b)** quantification of protein expression for the exosomal markers TSG101, Flotillin-1 and MFG-E8 in the culture media of DRG neurons incubated with HEPES buffer+glucose (1mg/ml) (CON) or with 25 mM KCl or 50 mM KCl for 3 hours. Data are means ± S.E.M., n=4 cultures; **P*<0.05 and ****P*<0.001, one-way ANOVA, post-hoc Bonferroni.

a

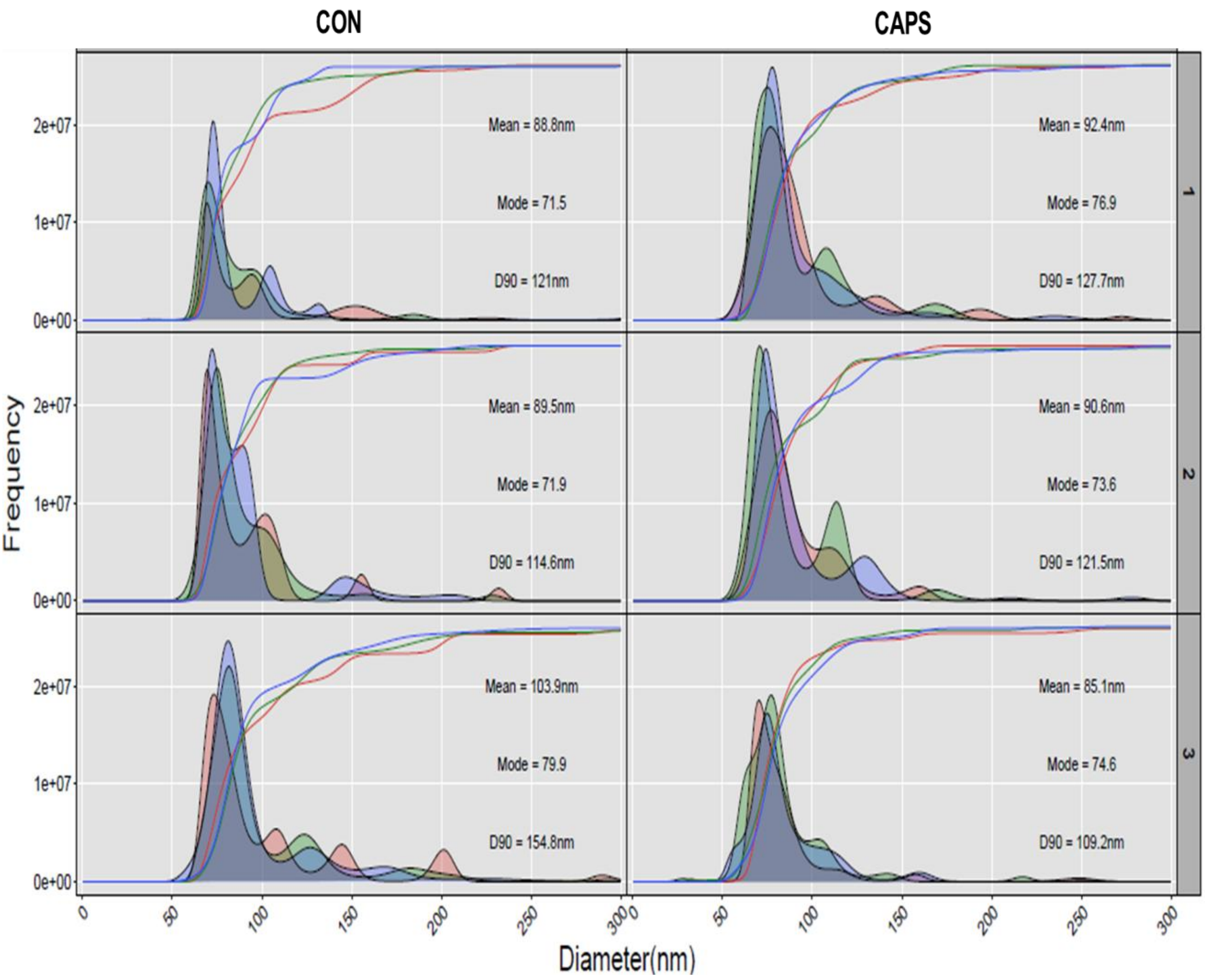

b

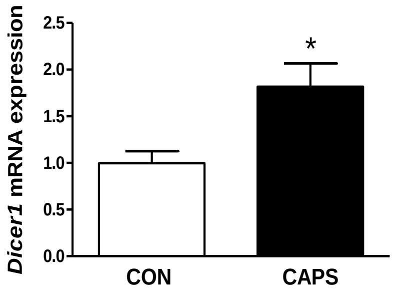

c

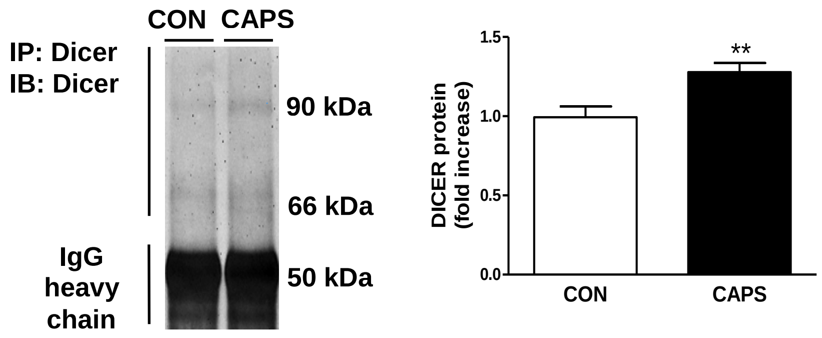

Supplementary Figure 4. Incubation of sensory neurons with capsaicin induces release of EVs including exosomes and an intracellular increase of Dicer. (a) Nanosight detection of exosomes isolated from culture media of neurons incubated with buffer control (CON) or CAPS for 3 hours. A triplicate for each condition is reported. The inset corresponds to the mean diameter and mode diameter and D90 values represent the

diameter of 90% of the particles. Exosomes levels were determined by the number of particles comprised between 30 nm and 120 nm. Expression of *Dicer* mRNA (**b**) and protein (**c)** in cultured DRG treated with control buffer or CAPS for 3 hours. Data are means ± S.E.M., n=6; **P*<0.05 and ***P*<0.01 compared to control, Student's *t* test.

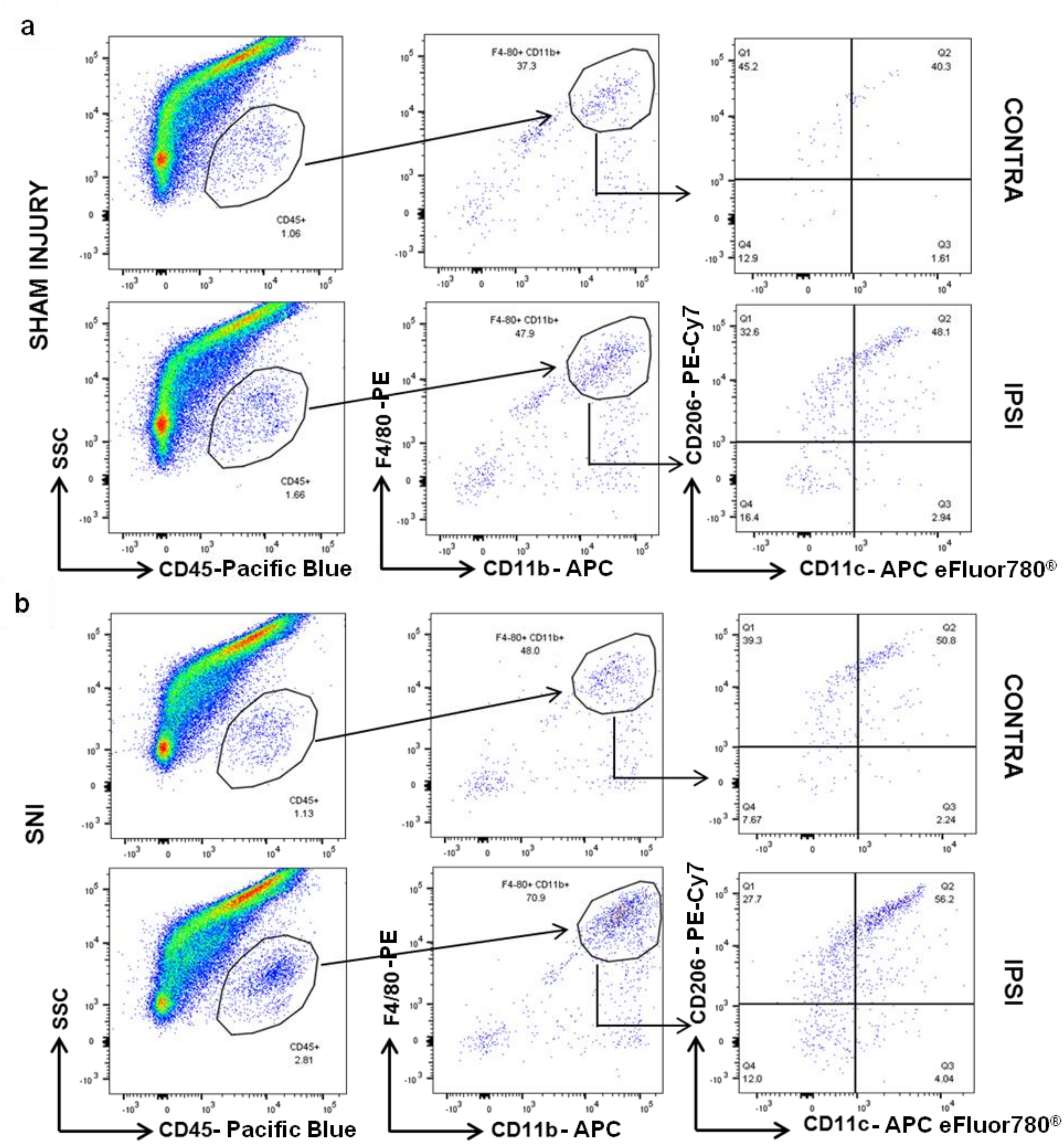

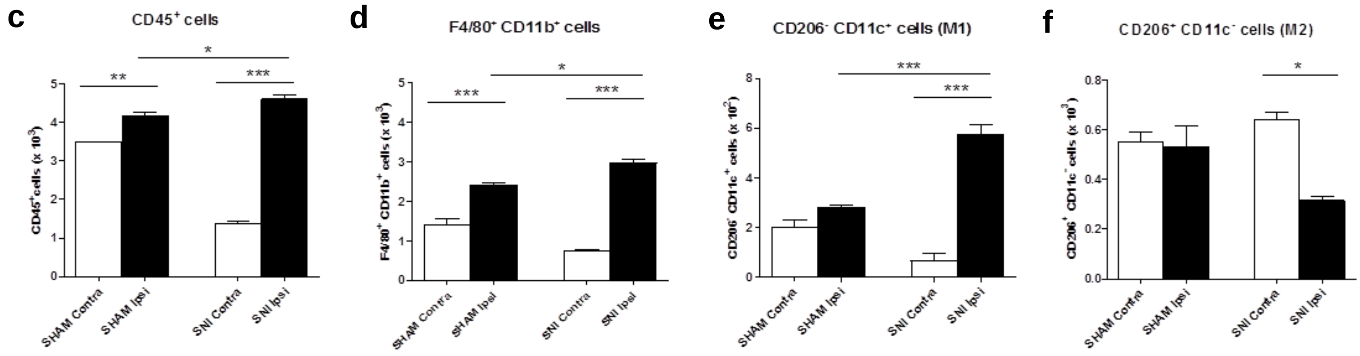

Supplementary Figure 5. Spared nerve injury induces an increase of immune cells recruitment in DRG. Representative scatterplots of immune cells sorted from pools of contralateral and ipsilateral L4 and L5 DRG obtained from SHAM injured **(a)** or SNI mice **(b).** Cells were gated on $CD45^+$, $F4/80^+$ and $CD11b^+$. Macrophages were defined as $CD11b^+$ $F4/80^+$ and were further analysed for the M2 ($CD206^+$ $CD11c^-$) and M1 ($CD206^-$ $CD11c^+$) phenotypes. Numbers in gates refer to percentage of positive cells for each specific marker. **(c)** Bar charts represent absolute number in DRG of leukocyte ($CD45^+$), **(d)** macrophages ($F4/80^+$ $CD11b^+$), **(e)** M1 macrophages ($CD206^-$ $CD11c^+$) and **(f)** M2 macrophages ($CD206^+CD11c^-$). Flow cytometry analysis was performed on data from 4 independent experiments. Data are expressed as means ± S.E.M., n=4 for each group. *P<0.05, **P<0.01 and ***P<0.001, one-way ANOVA, post-hoc Bonferroni.

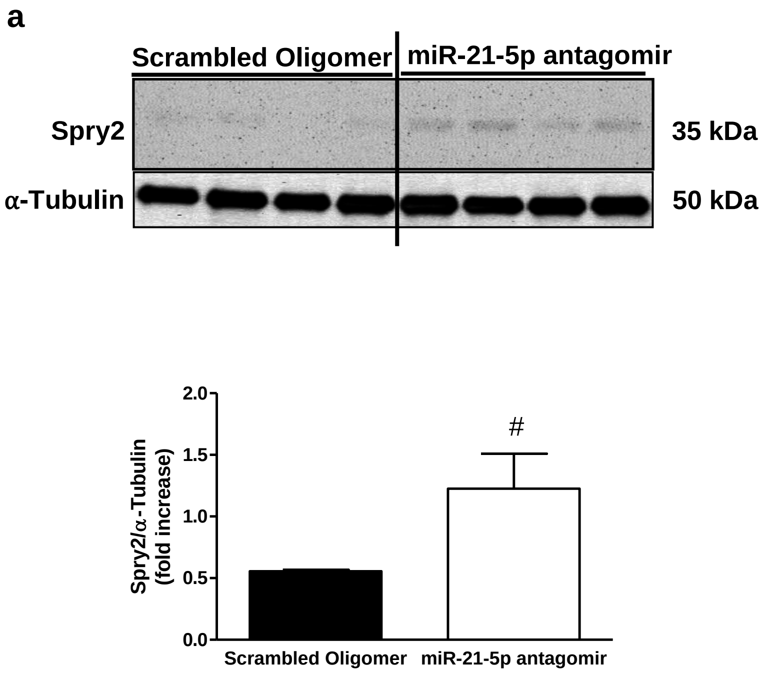

b

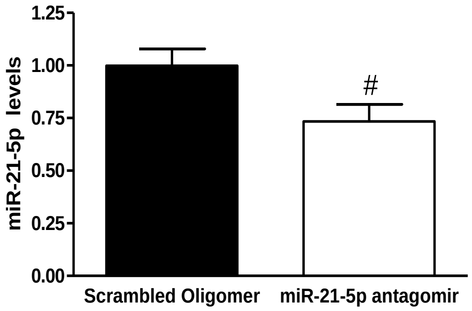

Supplementary Figure 6. Effect of intrathecal delivery of miR-21-5p antagomir and scrambled oligomer on Sprouty2 protein and miR-21-5p expression. Intrathecal cannulation and osmotic pump implantation were performed on day 0 and the delivery of miR-21-5p antagomir or scrambled control started on the same day. After 7 days, efficient delivery of miR-21-5p antagomir to L4 and L5 DRG was confirmed by up-regulation of Spry2 protein expression (**a**) and down-regulation of miR-21-5p levels (**b**). Data are expressed as means ± S.E.M., n = 3-4 mice for each group. #P<0.05 compared to scrambled oligomer, Student's t test.

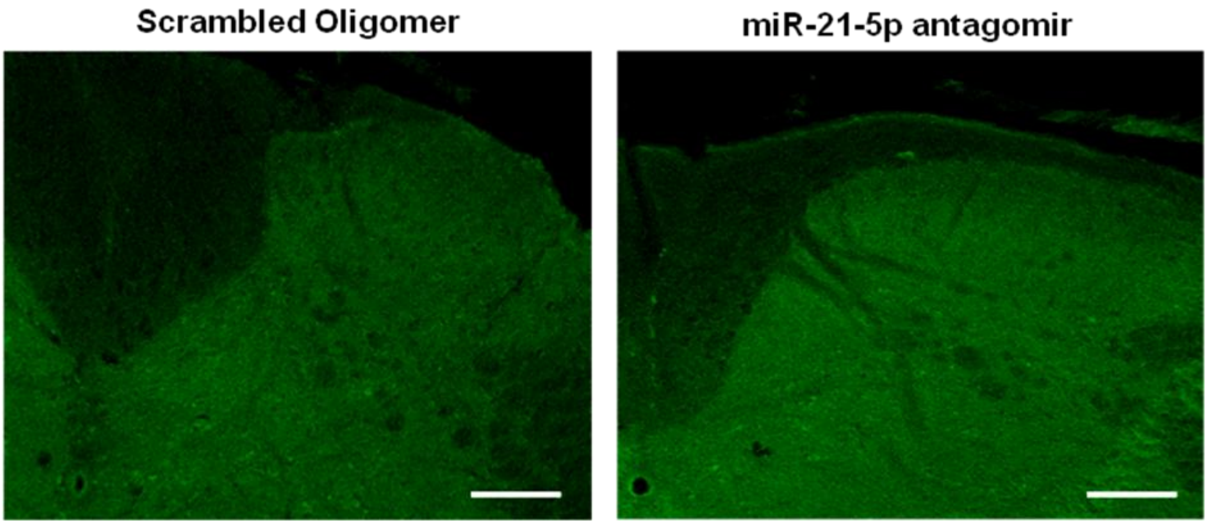

Supplementary Figure 7. miR-21-5p antagomir and scrambled oligomer are not retained by spinal cord neurons following intrathecal administration. Representative confocal images of cryo-sections of lumbar spinal cord taken from SHAM injured and SNI mice treated intrathecally for 7 days with miR-21 antagomir or scrambled oligomer. FAM signal was not detected. Scale bar represents 50 μm.

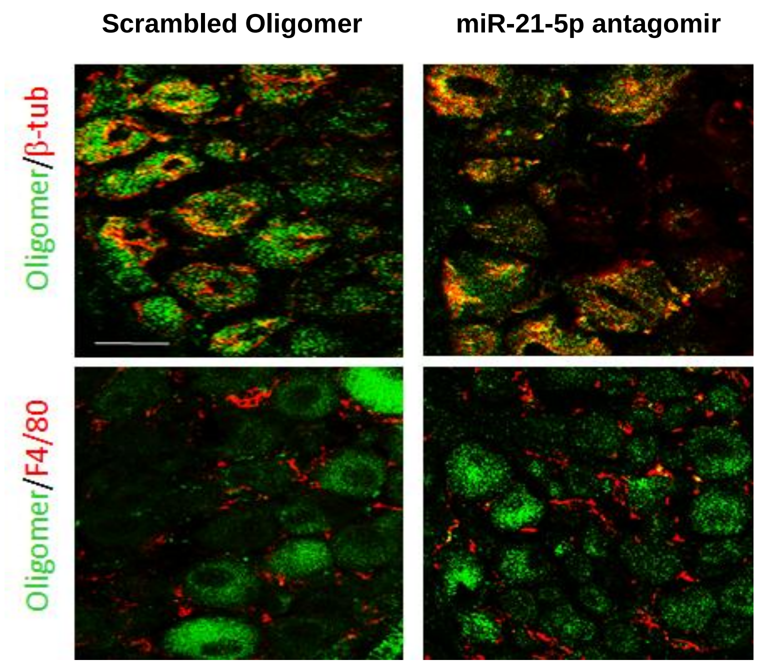

Supplementary Figure 8. Scrambled oligomer and miR-21-5p antagomir distribution in L4, L5 contralateral dorsal root ganglia. Both intrathecally-delivered scrambled oligomer and antagomir (green) co-localize with the neuronal marker β-tubulin (red) to a greater extent than macrophage marker F4/80^{+} (red). Scale bar= 50 µm.

a

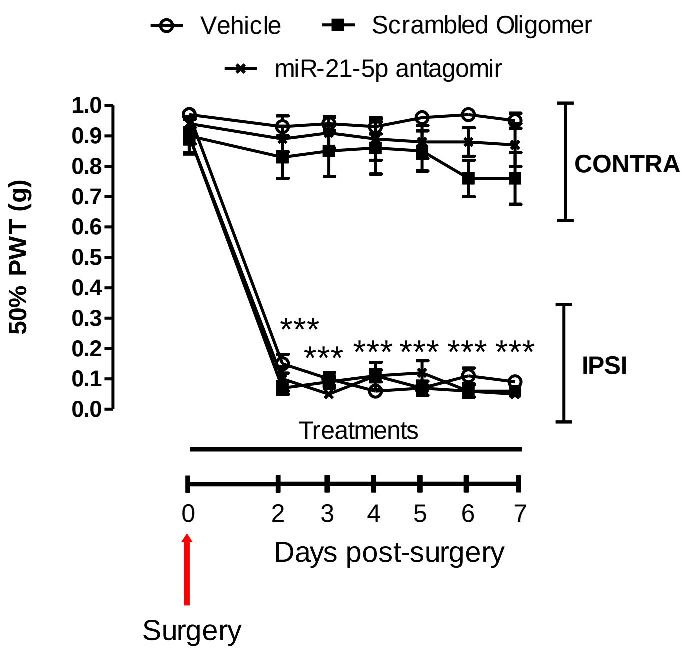

b

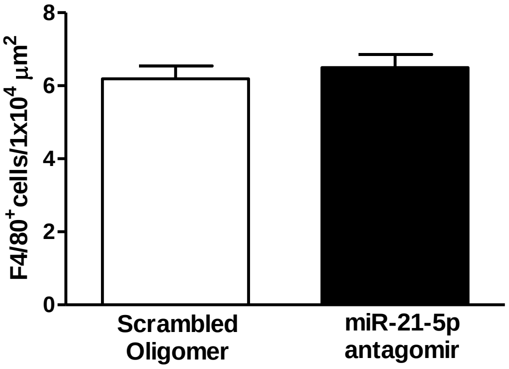

Supplementary Figure 9. Systemic delivery of miR-21 antagomir does not prevent mechanical hypersensitivity. (**a**) Effect of continuous subcutaneous delivery of the miR-21-5p antagomir (12 pmol/day) for 7 days on the development of mechanical hypersensitivity. No differences were observed between miR-21-5p antagomir, scrambled oligomer and vehicle groups. Data are presented as 50% of paw withdrawal thresholds (PWT); means ± S.E.M., n = 6 mice/group. ***P<0.001 compared to the corresponding contralateral paw, two-way ANOVA followed by Tukey test. (**b**) Quantification of F4/80$^+$ cells in L5 DRG ipsilateral to injury following systemic delivery of either the scrambled oligomer or miR-21-5p antagomir. Data are means ± S.E.M., n=4 mice/group.

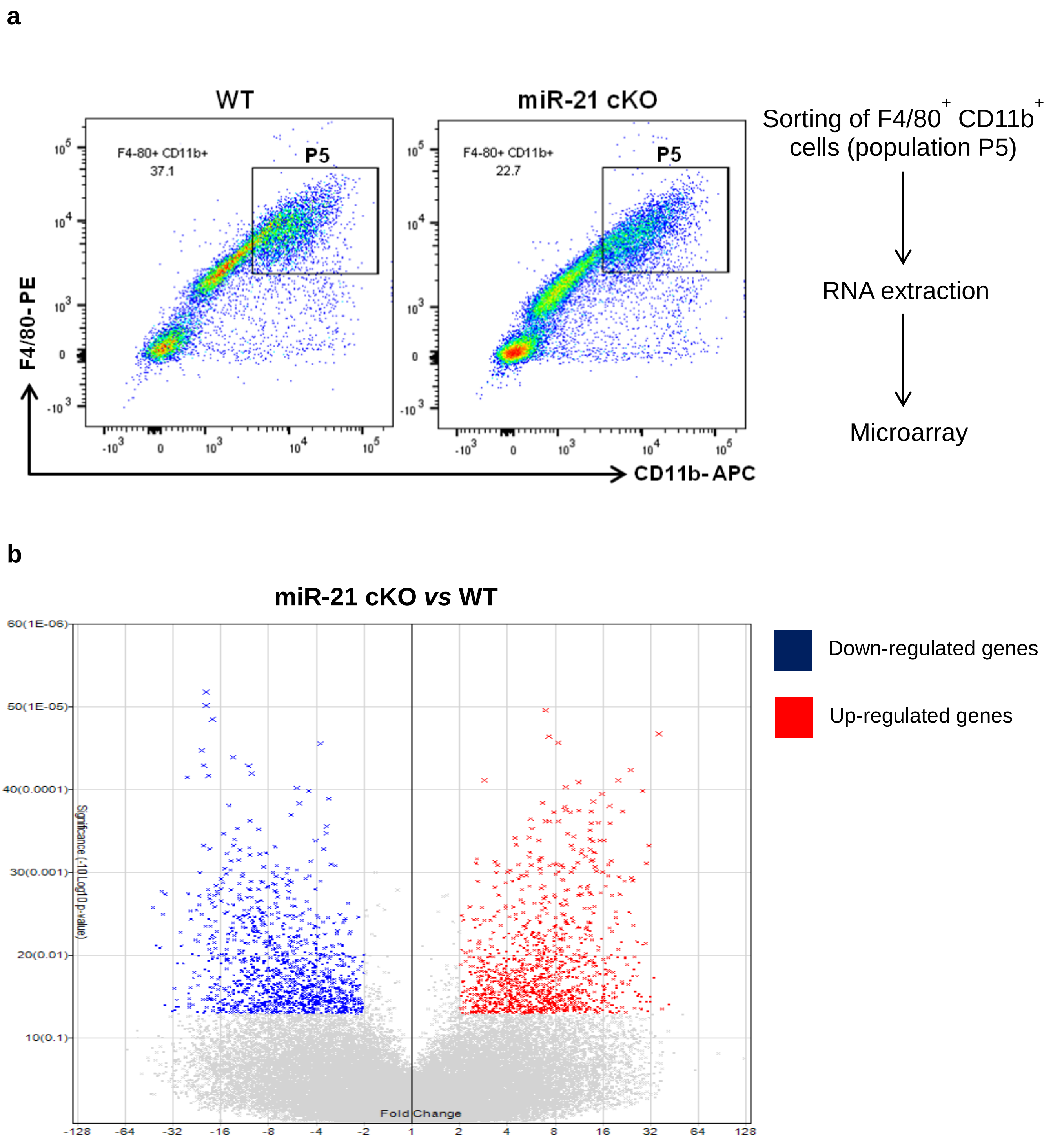
a
WT
miR-21 cKO
F4-80+ CD11b+
37.1
22.7
P5
F4/80- PE
CD11b- APC
Sorting of F4/80+ CD11b+ cells (population P5)
RNA extraction
Microarray
b
miR-21 cKO vs WT
Down-regulated genes
Up-regulated genes
Significance (-10 Log10 p-value)
Fold Change
60(1E-06)
50(1E-05)
40(0.0001)
30(0.001)
20(0.01)
10(0.1)
-128 -64 -32 -16 -8 -4 -2 1 2 4 8 16 32 64 128

c

Top 10 significant modified pathways

#	Maps	0 0.5 1 1.5 2 2.5 3 3.5 -log(pValue)	pValue
1	Signal transduction_Calcium signaling		3.899e-5
2	Apoptosis and survival_TNF-alpha-induced Caspase-8 signaling		1.128e-4
3	Cytoskeleton remodeling_Substance P mediated membrane blebbing		1.607e-4
4	Cytoskeleton remodeling_TGF, WNT and cytoskeletal remodeling		1.625e-4
5	Neurophysiological process_Receptor-mediated axon growth repulsion		1.694e-4
6	Regulation of lipid metabolism_RXR-dependent regulation of lipid metabolism via PPAR, RAR and VDR		1.985e-4
7	G-protein signaling_G-Protein alpha-12 signaling pathway		2.138e-4
8	NF-AT signaling in cardiac hypertrophy		2.541e-4
9	Apoptosis and survival_Ceramides signaling pathway		3.239e-4
10	Cytoskeleton remodeling_Role of PKA in cytoskeleton reorganisation		3.239e-4

d

**Known miR-21-5p Target Genes**			**cKO_vs_WT_Ipsi_P_0.05_FC_2_2**	**References (PMID):**
**Symbol**	**Full name**	**Object type**		
Acta2	Alpha-actin-2	Binding protein	2.73 fold	28515040;19561119
Tpm1	Tropomyosin-1	Binding protein	6.37 fold	19253296;17363372
Znf 288	Zinc finger protein 288	Transcription factor	12.69 fold	28707457

Supplementary Figure 10. Conditional deletion of miR-21 in sensory neurons is associated with gene changes in macrophages isolated from L4/L5 ipsilateral DRG. (**a**) Schematic strategy adopted to perform a microarray analysis (MA) on the F4/80$^+$ CD11b$^+$ macrophages (population P5) isolated from a pool of ipsilateral L4/L5 DRG in spared nerve injured WT and miR-21 cKO (n=5 mice/group). (**b**) MA plot of significance (-10 Log10 P-value) *vs* fold changes in miR-21 cKO macrophages (n=3 replicates) compared to WT macrophages (n=3 replicates). (**c**) List of top 10 significant regulated pathways in miR-21 cKO macrophages compared to WT macrophages analysed using a MetaCore™ software. (**d**) Table of known miR-21 target genes significantly upregulated in miR-21 cKO macrophages with a fold change cut-off of 2.2.

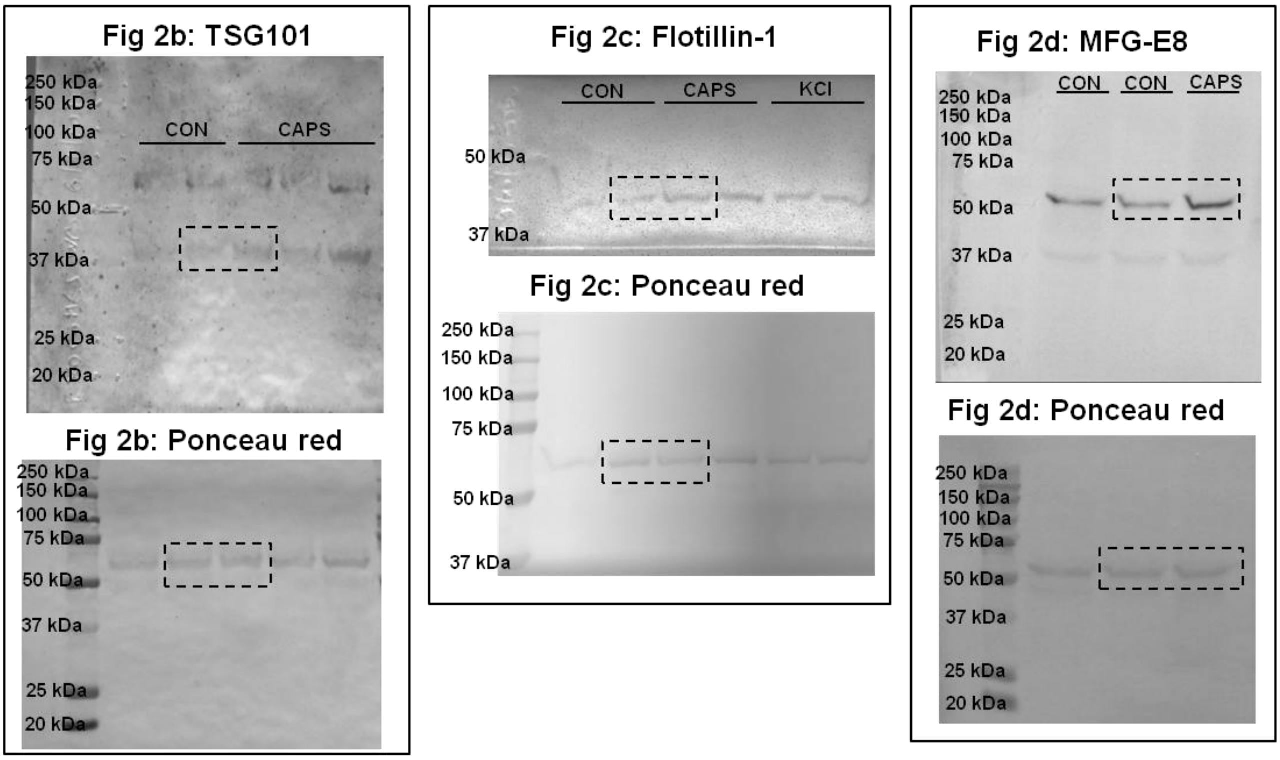
Fig 2b: TSG101
250 kDa
150 kDa
100 kDa
75 kDa
50 kDa
37 kDa
25 kDa
20 kDa
CON
CAPS
Fig 2b: Ponceau red
250 kDa
150 kDa
100 kDa
75 kDa
50 kDa
37 kDa
25 kDa
20 kDa
Fig 2c: Flotillin-1
CON
CAPS
KCl
50 kDa
37 kDa
Fig 2c: Ponceau red
250 kDa
150 kDa
100 kDa
75 kDa
50 kDa
37 kDa
Fig 2d: MFG-E8
CON
CON
CAPS
250 kDa
150 kDa
100 kDa
75 kDa
50 kDa
37 kDa
25 kDa
20 kDa
Fig 2d: Ponceau red
250 kDa
150 kDa
100 kDa
75 kDa
50 kDa
37 kDa
25 kDa
20 kDa

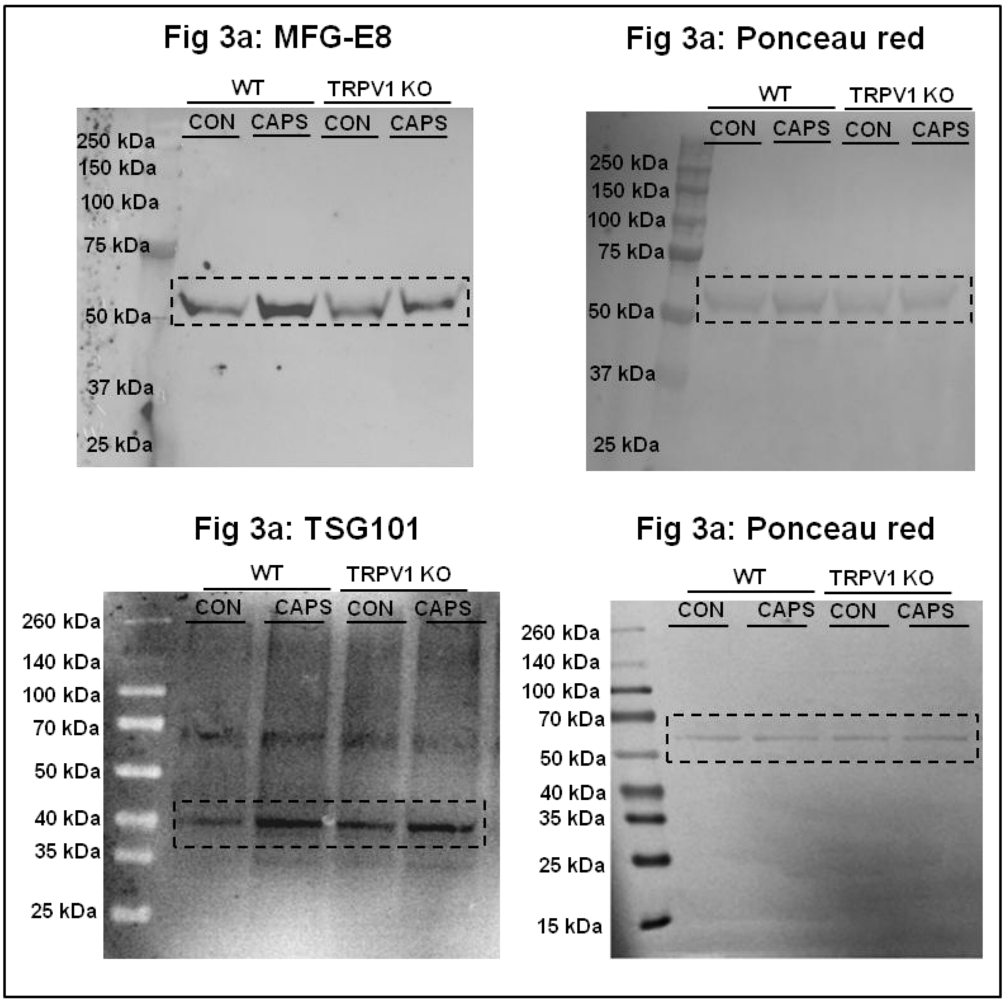
Fig 3a: MFG-E8
WT
TRPV1 KO
CON
CAPS
CON
CAPS
250 kDa
150 kDa
100 kDa
75 kDa
50 kDa
37 kDa
25 kDa
Fig 3a: Ponceau red
WT
TRPV1 KO
CON
CAPS
CON
CAPS
250 kDa
150 kDa
100 kDa
75 kDa
50 kDa
37 kDa
25 kDa
Fig 3a: TSG101
WT
TRPV1 KO
CON
CAPS
CON
CAPS
260 kDa
140 kDa
100 kDa
70 kDa
50 kDa
40 kDa
35 kDa
25 kDa
Fig 3a: Ponceau red
WT
TRPV1 KO
CON
CAPS
CON
CAPS
260 kDa
140 kDa
100 kDa
70 kDa
50 kDa
40 kDa
35 kDa
25 kDa
15 kDa

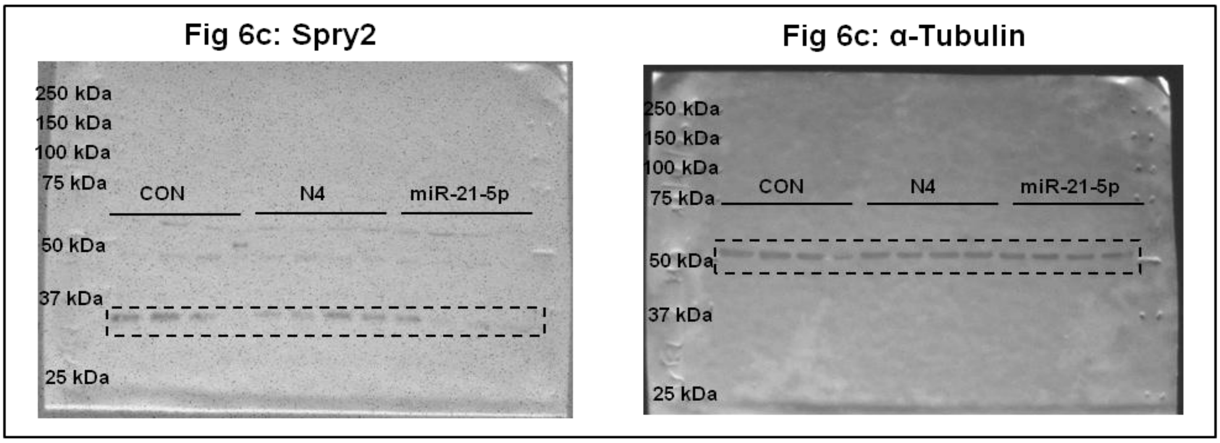

Fig 6c: Spry2
250 kDa
150 kDa
100 kDa
75 kDa
CON
N4
miR-21-5p
50 kDa
37 kDa
25 kDa
Fig 6c: α-Tubulin
250 kDa
150 kDa
100 kDa
75 kDa
CON
N4
miR-21-5p
50 kDa
37 kDa
25 kDa

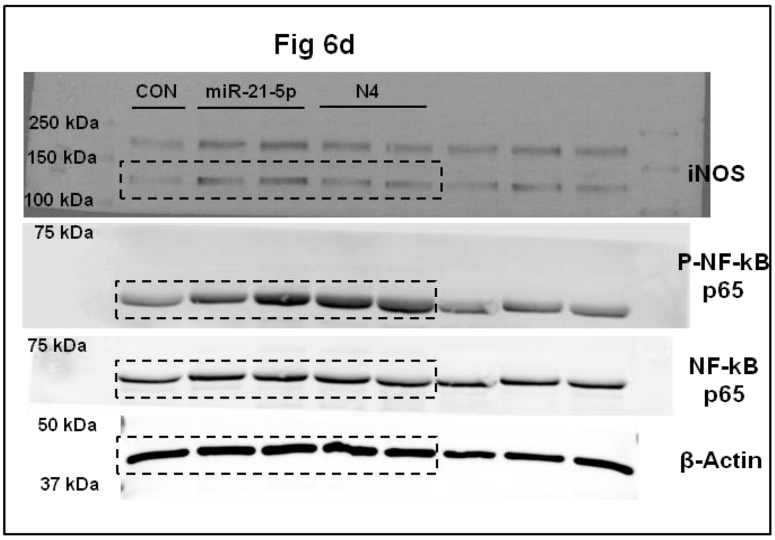

Fig 6d
CON
miR-21-5p
N4
250 kDa
150 kDa
100 kDa
iNOS
75 kDa
P-NF-kB p65
75 kDa
NF-kB p65
50 kDa
β-Actin
37 kDa

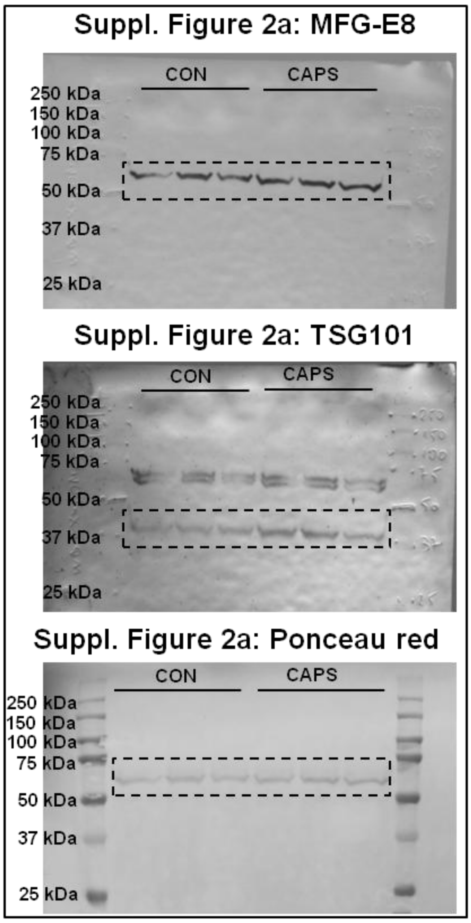

Suppl. Figure 2a: MFG-E8
CON
CAPS
250 kDa
150 kDa
100 kDa
75 kDa
50 kDa
37 kDa
25 kDa
Suppl. Figure 2a: TSG101
CON
CAPS
250 kDa
150 kDa
100 kDa
75 kDa
50 kDa
37 kDa
25 kDa
Suppl. Figure 2a: Ponceau red
CON
CAPS
250 kDa
150 kDa
100 kDa
75 kDa
50 kDa
37 kDa
25 kDa

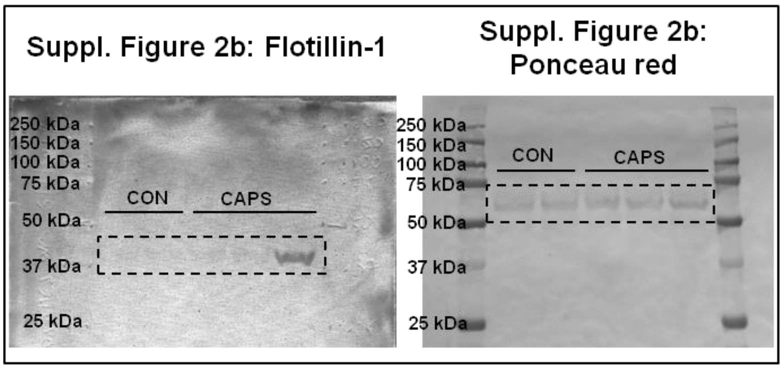

Suppl. Figure 2b: Flotillin-1
250 kDa
150 kDa
100 kDa
75 kDa
CON
CAPS
50 kDa
37 kDa
25 kDa
Suppl. Figure 2b: Ponceau red
250 kDa
150 kDa
100 kDa
75 kDa
CON
CAPS
50 kDa
37 kDa
25 kDa

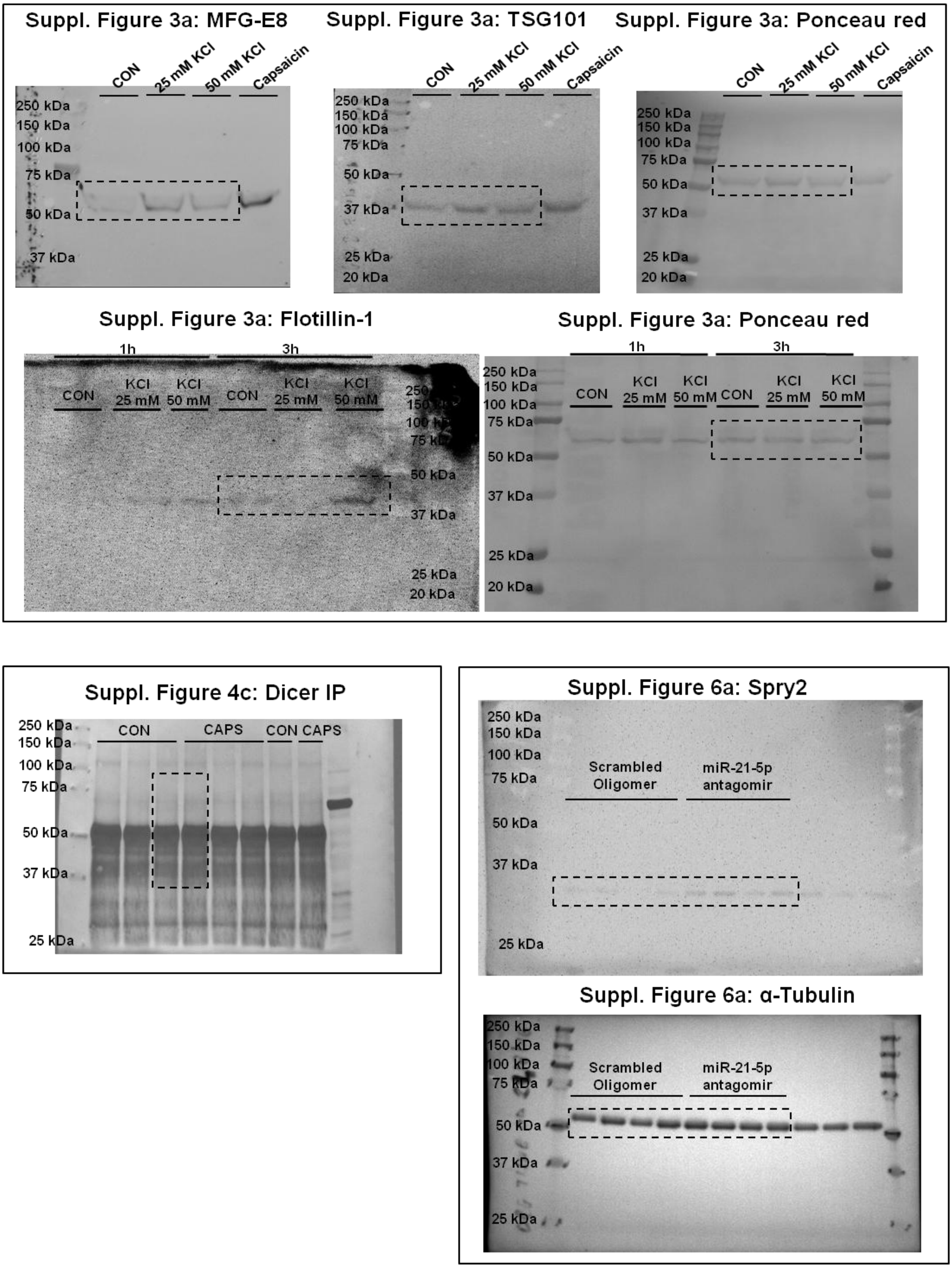

Supplementary Figure 11. Uncropped images of Western blot figures shown in the main paper and the Supplementary Figures.
